# Long-term outcomes of cable-suspended suture technique versus conventional suture for anterior vaginal wall prolapse: a retrospective cohort study

**DOI:** 10.1186/s12905-023-02228-z

**Published:** 2023-02-16

**Authors:** Qian Hu, Shuai Huang, Xiaoke Yang, Ye Li, Qiubo Lv

**Affiliations:** 1 Department of Obstetrics and Gynecology, Beijing Hospital, No. 1 Dahua Road, Dongcheng District, Beijing, 100730 People’s Republic of China; 2 National Center of Gerontology, National Health Commission, Beijing, People’s Republic of China; 3 Institute of Geriatric Medicine, Chinese Academy of Medical Sciences, Beijing, People’s Republic of China

**Keywords:** Anterior vaginal wall prolapse, Transvaginal surgery, Cable-suspended suture

## Abstract

**Background:**

Anterior colporrhaphy (AC) is a conventional surgical repair technique for cystocele but with high recurrence rate. We present a novel technique: Cable-suspended structure (CSS) by non-absorbable suture combined with "bridge" formation in surgical treatment of cystocele. This study aimed to evaluate and compare the long-term outcome of CSS technique for anterior vaginal wall repair with AC.

**Methods:**

A retrospective review was performed on patients who underwent anterior vaginal wall repair between January 2012 and March 2017 at our center. All the patients were under a follow-up survey. The primary outcomes were objective cure (anterior prolapse POP-Q ≤ stage 1) and subjective cure (no symptoms of bulge or retreatment for prolapse). Secondary outcomes included quality of life (QOL) and patients’ satisfaction, outcomes of site-specific POP-Q points Aa, Ba and C, as well as postoperative complications.

**Results:**

Of 91 included participants, 43 underwent AC and 48 underwent CSS. The proportion of sarcrospinous ligament fixation in the CSS group was higher than in the AC group (81.4% vs. 77.1%, *P* < 0.05). At a median follow-up of 69 months, the CSS group showed significantly higher objective cure rate compared with the AC group (72.9% vs. 51.2%, odds ratio 2.57, 95%CI 1.07–6.16). After adjusting for sarcrospinous ligament fixation, the CSS group still significantly showed higher objective cure rate (adjusted odds ratio 2.88, 95%CI 1.16–7.21). The proportion of the patients with POP-Q 0 stage in the CSS group was particularly higher than the AC group (25% vs. 7.0%, *P* = 0.025). There was no difference between the groups with respect to subjective cure, patients’ satisfaction and postoperative complication.

**Conclusions:**

The CSS technique showed better objective outcome than AC, however, subjective cure rate did not significantly differ between the two. Future prospective trial with large-scale should confirm the effectiveness and safety of CSS in sexually active women.

## Introduction

Pelvic organ prolapse (POP) is a common disease that affects more than 40% of women aged over 50 years worldwide, and its incidence keeps increasing due to development of global aging [1]. Prolapse of the anterior vaginal wall is one of the most common types of POP with high recurrence rate. Various minimally invasive repair operations have been widely used in clinical treatment, and among which vaginal surgery is the most common owing to the minimal trauma and high cost-effectiveness [2].

Native tissue repair and mesh repair are typical surgical treatment strategies for anterior vaginal wall prolapse. Currently, native tissue repair is the first-choice treatment, while the recurrence rate is high and the long-term repair durability is poor, especially for patients with severe prolapse [3, 4]. Although the application of synthetic mesh in anterior compartment was shown to be superior to native tissue repair for surgical outcome [5], concerns over mesh-related complications and the high rate of re-operation has limited their use in vaginal repair [6]. To the best of our knowledge, there is still no consensus on the best surgical treatment strategy for anterior vaginal prolapse.

Since 2007, we have adopted a novel non-mesh technique, cable-suspended sutures (CSS) overlying a bridge-like vaginal support structure, to treat anterior vaginal wall prolapse, aiming to improve the efficacy while minimizing surgical complications [7]. The purpose of this study is to present the long-term effectiveness and safety of CSS and compare with that of conventional non-mesh anterior colporrhaphy (AC).

## Methods

### Patient and data collection

This was a retrospective cohort study. We reviewed charts of consecutive patients who underwent anterior vaginal repair for anterior vaginal wall prolapse between January 2012 and March 2017 at Beijing Hospital. For the comparison study, the inclusion criteria were defined as follows: (1) hospitalized patients with primary pelvic organ prolapse quantification (POP-Q) stage 3 of anterior vagina; (2) surgical correction with anterior colporrhaphy or CSS technique; (3) postoperative follow-up for at least five years. And the exclusion criteria were: (1) patients who have uterine preservation; (2) patients with incomplete data; (3) patients underwent prior hysterectomy.

The primary outcomes were the objective cure rate of POP, defined as anterior prolapse of POP-Q ≤ stage 1, and the subject cure rate defined as answering “No” to question 3 of the Pelvic Floor Distress Inventory (PFDI-20) and the absence of any reoperation for POP [8]. Secondary outcomes include quality of life (QOL) and patients’ satisfaction, outcomes of site-specific POP-Q points Aa, Ba and C, and postoperative complications (scored according to the Clavien-Dindo classification of surgical complications) [9].

Chart review including detailed medical history, urogenital examination, urine analysis, and documented follow-up were performed. The degree of genital prolapse was assessed using the POP-Q System [10]. Validated Chinese versions of the questionnaires, including the short form of PFDI-20 and the Pelvic Floor Impact Questionnaire (PFIQ-7), were used to evaluate functional results of the patients [11, 12].

In our institution, the patients after discharge were scheduled for follow-up visits at 1, 3, 6, 12 months postoperatively and annually thereafter. Each visit included POP symptoms, pelvic examination and POP-Q evaluation. To assess the long-term effect of the techniques, postoperative evaluation at the last follow-up visit was included. For the patients with recurrent prolapse who underwent repeat surgery, assessment on the last visit prior to the second surgery was used in analysis. To evaluate the patient’s personal satisfaction, the following questions were asked at their last visit (1) “Are you satisfied with the surgical procedure?” (2) “would you go for the same choice during the counseling before surgery?”.

### Surgical procedure

All patients were counseled during surgical management and were informed of any potential efficacy and complications from the treatment. Hysterectomy was performed for patients who required uterine removal via vaginal approach. Unilateral sarcrospinous ligament fixation (SLF) was performed to the right sacrospinous ligament for patients who needed apical suspension [13]. Kelly plication sutures had been used for patients with stress urinary incontinence (SUI), if no tension free vaginal tape (TVT) procedure was planned. Other procedures were performed where necessary.

All the surgical procedures were performed by experienced surgeons, according to the procedure as follows:

The patients were placed in dorsal lithotomy position, and a Foley catheter was inserted. After a hysterectomy, an adequate volume of normal saline was injected under the anterior vaginal epithelium in an accurate plane before dissection.

AC was performed by making a midline, sagittal incision in the anterior wall of the vagina from the level of the vaginal cuff to a point 1.5 cm proximal to the external urethral meatus. The vaginal epithelium was dissected away from the underlying anterior pubocervical connective tissue using sharp dissection. This dissection was continued laterally to the level of the descending ischiopubic rami on both sides. The pubocervical connective tissue was then plicated in the midline using a series of interrupted, delayed absorbable sutures.

**Fig. 1:**
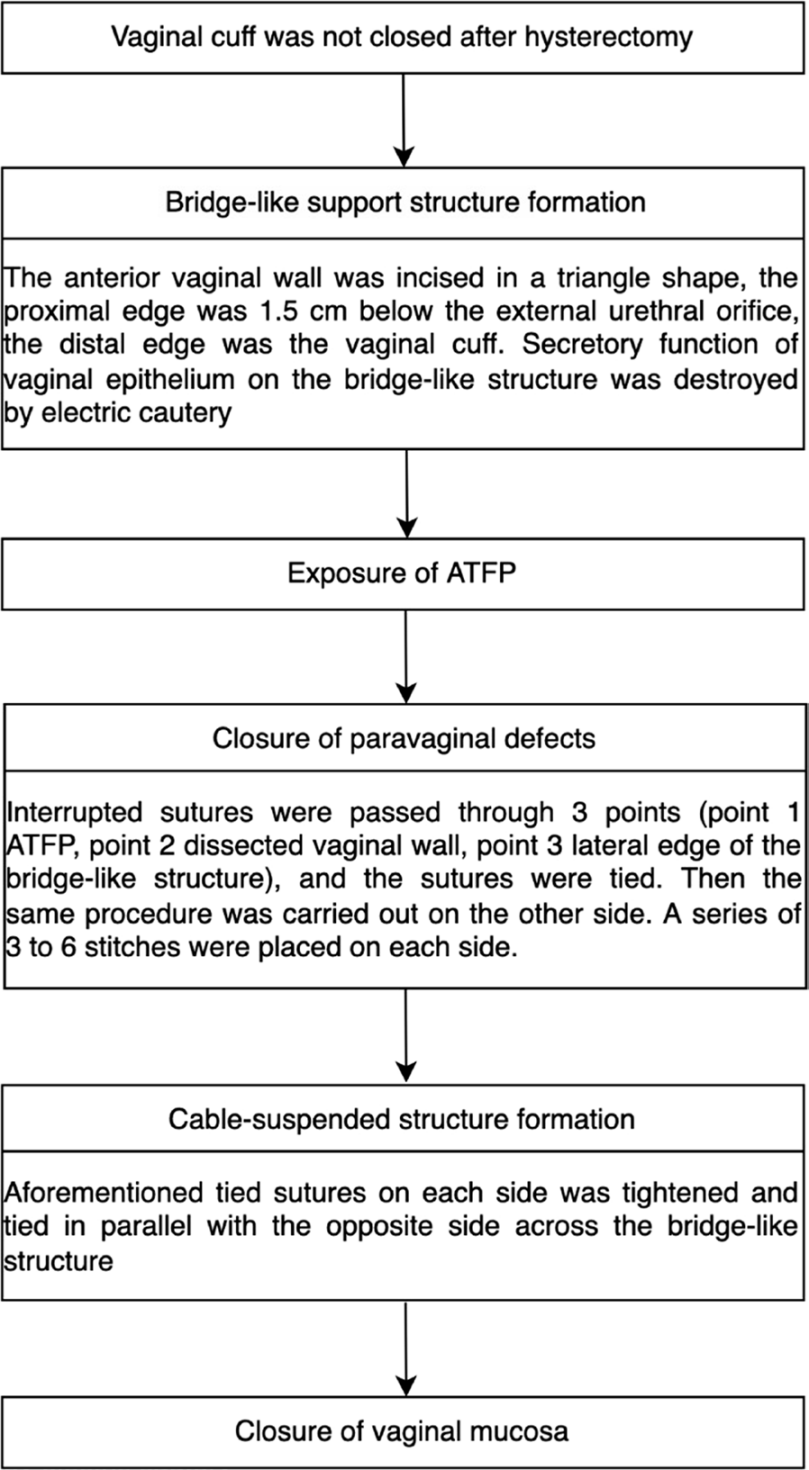
The flow diagram for CSS procedure

CSS technique followed as our previous protocol [7]. Briefly, a triangle-shaped incision of the anterior vaginal wall was performed from the bladder neck to the vaginal cuff, and the vaginal epithelium of the triangle-shaped region, which was destroyed by electric cautery, was retained as a bridge-like support structure. Dissection of the vaginal flap was performed laterally to the arcus tendinous fascia pelvis (ATFP). From the level of the bladder neck to proximally above the ischial spine, interrupted stitches with a non-absorbable suture were placed at three points on each side, which located on the ATFP, vaginal muscularis or pubocervical fascia and lateral edge of the triangle-shaped epithelium. Then the lateral suture at aforementioned three points was tightened on each side and tied in parallel with the opposite side across the bridge-like structure. Finally, the vaginal mucosa was trimmed and then closed using a running, locking absorbable suture in both procedures. The flow diagram for CSS procedure is shown in Fig. 1.

### Statistical analysis

Data were analyzed using SPSS IBM 23.0 (SPSS, Inc., Chicago, IL). Continuous variables were shown as mean ± standard deviation (SD) or as median and interquartile range (IQR), and categorical variables were shown as number count and percentage. Descriptive statistics were calculated for demographics and perioperative data. Categorical variables were analyzed using the chi-square or Fisher’s exact test. Continuous data were analyzed using t-test or Mann–Whitney U-tests. Logistic regression analysis was used to obtain odds ratios (OR) with 95% confidence interval (CI) of cure for CSS group compared with AC group. *P* < 0.05 was considered statistically significant.

## Results

Over the timeframe of this study 91 patients were included, in which 48 patients in the CSS group and 43 in the AC group. The list of demographic and clinical characteristics of both groups is shown in Table 1. The mean patient age was 67.3 years in the AC group and 67.6 years in the CSS group. With the exception of SLF, there were no significant differences in the patient characteristics at baseline between the two groups. For all patients had a concomitant procedure performed, operating time for the specific anterior procedure could not be separated from the total operating time and have not been specifically described here.

**Table 1:** Patient characteristics

Characteristic	AC Group (*n* = 43)	CSS Group (*n* = 48)	*P* value
Age (years), mean (SD)	67.3 (7.7)	67.6 (7.7)	0.954
BMI (kg/m^2^), mean (SD)	24.9 (3.5)	25.7 (1.8)	0.192
Parity, median (IQR)	2 (1.5)	2 (2.5)	0.094
SUI, *n* (%)	15 (34.9)	13 (27.1)	0.421
Preop anterior POP-Q stage 3, *n* (%)	43 (100)	48 (100)	–
Preop point Aa (cm), mean (SD)	2.1 (1.3)	2.6 (0.6)	0.234
Preop point Ba (cm), mean (SD)	3.2 (1.7)	3.3 (1.1)	0.820
Preop point C (cm), mean (SD)	0.8 (1.7)	1.2 (1.1)	0.191
Concomitant procedures			
TVH, *n* (%)	43 (100)	48 (100)	–
SLF, *n* (%)	35 (81.4)	37 (77.1)	< 0.001*
Posterior repair, *n* (%)	6 (14.0)	11 (22.9)	0.251
TVT, *n* (%)	1 (2.3)	2 (4.2)	1.000
Follow-up (months), median (IQR)	67 (12)	69.5 (10.75)	0.799

Data are presented as *n* (%) unless otherwise stated. *P < 0.05 for all characteristics. Independent samples *t* test for continuous variables, Chi-square test or Fisher’s exact test for categorical variables. *SD*, standard deviation, *IQR* interquartile range, *BMI* body mass index, *SUI* stress urinary incontinence, *TVH* transvaginal hysterectomy, *SLF* sacrospinous ligament fixation, *TVT* tension-free vaginal tape

### Primary outcomes

Table 2 presents primary outcomes. At median follow-up of 67 months for the AC group and 69.5 months for the CSS, objective cure rate was significantly higher in CSS group than in the AC group (72.92% vs. 51.16%, *P* = 0.032). Both groups showed high subjective cure rates, 87.5% for CSS group and 79.07% for the AC group, but with no significant difference (*P* = 0.379). In unadjusted analysis, the odds of the objective cure were higher in the CSS group compared to the AC group (OR 2.57, 95%CI 1.07–6.16). After adjusted for SLF, a significant difference was again noted (adjusted OR 2.88, 95% CI 1.16–7.21). In both crude and adjusted analyses, CSS did not significantly improve the subjective cure of patients with anterior vaginal prolapse compared with AC (OR 1.85, 95% CI 0.6–5.72 vs. adjusted OR 2.32, 95% CI 0.67–7.99) (Table 3).

**Table 2:** Primary outcome and postoperative complications

Outcome	AC Group (*n* = 43)	CSS Group (*n* = 48)	*P* value
Objective cure, *n* (%)	22 (51.2)	35 (72.9)	0.032*
Subjective cure, *n* (%)	34 (79.1)	42 (87.5)	0.379
Surgery for prolapse recurrence	1 (2.3)	1 (2.1)	–
Clavein-Dindo, *n* (%)	2 (4.7)	4(8.3)	–
Grade I			
Pelvic pain	0	1 (2.1)	–
Squamous epithelial vaginal inclusion cyst	0	1 (2.1)	–
Physicotherapy for SUI	1 (2.3)	0	–
Grade II			
Infection	1 (2.3)	0	–
Grade III	0	0	–
Grade IV	0	0	–

Data are presented as *n* (%). Fisher’s exact test for all. *P < 0.05 for all characteristics

**Table 3:** Odds ratios for the cure rates

	Objective cure OR (95%CI)	Subjective cure OR (95%CI)
Crude	2.57 (95%CI 1.07, 6.16)	1.85 (95%CI 0.60, 5.72)
Adjusted for SLF	2.88 (95%CI 1.16, 7.21)	2.32 (95%CI 0.67, 7.99)

*OR* odd ratio, *CI* confidence interval, *SLF* sacrospinous ligament fixation

### Secondary outcomes

There were no severe surgical complications for either group (Table 2). One patient in CSS group had mild and paroxysmal pelvic pain, which was resolved with no intervention. Squamous Epithelial Inclusion Cysts was detected in one patient from the CSS group during follow-up without any symptom. Urinary tract infection presented in one patient in the AC group, which was managed conservatively with antibiotics.

During the follow-up period, one patient (2.3%) in the AC group and one (2.1%) in the CSS group experienced recurrence of prolapse. They both had recurrence in the anterior vaginal wall and vaginal vault and underwent sacrocolpopexy only.

Table 4 displays the changes of POP-Q stages including Aa, Ba and C point measurements. Fourteen out of 48 patients in the CSS group had stage 2 to 4 anterior vaginal wall prolapse, and twenty-one out of 43 patients in the AC group. Compared with the AC group, more patients had stage 0 anterior prolapse in the CSS group postoperatively (25% vs. 7.0%, *P* = 0.025). After surgery, anatomical measures of points Aa, Ba and C improved in both groups, but there were no significant differences between the groups.

**Table 4:** Postoperative improvement on POP-Q

	AC Group (*n* = 43)	CSS Group (*n* = 48)	*P* value
POP-Q stage, *n* (%)			
Stage 0	3 (7.0)	12 (25.0)	0.025*
Stage 1	19 (44.2)	23 (47.9)	0.722
Stage 2	14 (32.6)	5 (10.4)	0.011*
Stage 3	6 (14.0)	9 (18.8)	0.538
Stage 4	1 (2.3)	0 (0)	0.473
Point Aa (cm), mean (SD)	− 1.3 ± 1.8	− 1.9 ± 1.7	0.126
Point Ba (cm), mean (SD)	− 0.7 ± 1.7	− 1.1 ± 1.8	0.117
Point C (cm), mean (SD)	− 3.2 ± 2.1	− 3.0 ± 2.4	0.933

Fisher’s exact test for POP-Q stage, independent *t* test for point Aa, Ba and C. *P < 0.05 for all characteristics

The results from the quality of life questionnaires are listed in Table 5. Both groups showed improvement in the PFDI-20 and PFIQ-7 scores, although there were no significant differences between them. Regarding the Patient’s satisfaction, 37 patients (86.1%) in the AC group and 45 (93.8%) in the CSS group were satisfied with outcome of the surgery. Most patients in both groups did not regret their original choice of the surgical procedure (93% vs. 95.8%, *P* = 0.664).

**Table 5:** Subject outcomes by surgery type

	AC Group (*n* = 43)	CSS Group (*n* = 48)	*P* value
Change from baseline			
PFDI-20, median (IQR)	− 22.91 (38.54)	− 30.21 (34.64)	0.642
PFIQ-7, median (IQR)	− 42.86 (47.61)	− 38.1 (33.34)	0.883
Patient Satisfaction, *n* (%)	37 (86.1%)	45 (93.8%)	0.298
Patient confirm the same choice, *n* (%)	40 (93.0%)	46 (95.8%)	0.664

Mann–Whitney *U* test for questionnaire scores and Fisher’s exact test for others. *P < 0.05 for all characteristics. *IQR* interquartile range; *PFDI-20* pelvic floor distress Inventory, *POPDI* Pelvic Organ Prolapse Distress Inventory

## Discussion

Anterior compartment prolapse remains a challenging aspect of pelvic reconstructive surgery for gynecologists. To reduce the high recurrence rate of native tissue repair and the high incidence of mesh related complications, we proposed an effective and non-mesh approach for treating anterior vaginal prolapse.

Anterior colporrhaphy is the most widely used surgical treatment for anterior vaginal prolapse, but the success rate has been reported to be as low as 30% [14]. In this study, the objective cure rate was 58%, which is slightly higher than in previous studies. It might be associated with SLF surgery performed simultaneously. Since ATFP has been considered as the key supporting structure of anterior pelvic cavity, paravaginal repair was used to repair the lateral defect caused by tears between vaginal wall and lateral fascia. Some studies suggested that the success rate of paravaginal repair is relatively high, ranging from 55 to 89% [15, 16]. However, Weber and his colleagues found that there were no significant differences in anatomic success rate and symptom improvement between AC and paravaginal repair groups in a prospective randomized trial [17]. So far, there is no sufficient evidence to support which type of surgical procedures could provide the best outcome for patients with anterior vaginal prolapse.

The weakness of native tissue and its property of deteriorating with time will increase the risk of recurrence. To solve this problem, the CSS technique was proposed to improve the efficacy and durability of anterior repair by combining advantages of native tissue and non-absorbable sutures. In this study, the 72.9% long-term anatomic cure rate for CSS is higher than previously reported clinical series [16]. Based on paravaginal repair, the CSS uses ATFP as fixed structure, but has two distinctive characteristics. First, a triangle-shaped vaginal epithelium at the midline of anterior vaginal wall is used as a bridge-like structure to provide suspension by its fixation to ATFP. Cosson et al. presented a vaginal patch plastron technique for the treatment of cystocele that is similar to ours. Their result demonstrated that 93% of patients attained anatomical cured with 16 months follow-up, which is consistent with 94.8% of our early results [18]. However, we could not find subjective evaluation and long-term results of this approach. Secondly, a cable-suspended structure composed of non-absorbable sutures was used to reinforce the durability. Lee U et al. used interlocking permanent prolene sutures to provide a net of support for the correction of anterior vaginal prolapse, it presented a significant symptomatic improvement and quality of life promotion, but complications including suture exposure and ureteric obstruction were also noted [19]. During the initial application of CSS, prolene sutures were also be used. Although no suture exposure occurred, several patients complained of pain or discomfort especially during sex, so silk sutures with good knot security were used instead.

Pelvic floor prolapse is a complex condition, which contains not only structure defect but also dysfunction. So subjective feeling is very important for evaluation of pelvic floor reconstructive surgery. 87.5% of patients in the CSS group achieved subjective cure during the follow-up period. Additionally, a comparison between the two groups presented no significant difference among them (*P* = 0.379), which indicates CSS does not increase patients’ discomfort as other procedures with non-absorbable materials.

Apical defect is a risk factor for the recurrence of anterior vaginal prolapse [20]. In this study, neither of the patients with recurrent prolapse underwent vaginal apical suspension in the primary surgery. Both patients underwent sacrocolpopexy with no recurrence at last visit. Sacrocolpopexy has been the gold standard procedure for apical pelvic organ prolapse. However, this mesh-based form is associated with complications, such as erosion, infection and pain [21]. Transvaginal no-mesh surgery, such as sacrospinous ligament fixations and uterosacral ligament suspension (USLS), is preferred option in our center. The CSS technique as a transvaginal operation can perform with apical suspension simultaneously, and provides an individualized treatment option.

Patients underwent CSS in our center had a low rate of complications (4.2%), and these were mainly pelvic pain and squamous epithelial vaginal inclusion cyst. Epithelial vaginal inclusion cyst is a specific risk of CSS technique, and the management depends on symptoms of the patient. Cases without obvious symptoms can be observed. If patients complain of discomfort, resection of the cyst could be considered. To prevent the epithelial vaginal inclusion cyst, the secretory function of the triangle-shaped vaginal epithelium must be destroyed completely. Furthermore, two cases of newly appearing SUI in the CSS group were observed, and one (2.1%) required subsequent anti-incontinence surgery. Subsequent surgery for de novo SUI following vaginal prolapse repair has been reported as 3.6% [22]. Thus, we advocate for a staged approach and cautiously offered concomitant incontinence surgery.

The advantages of this study are the long duration of follow-up, the use of POP-Q assessment and validated questionnaires for measurement of subjective outcomes. To increase the validity of the data, we had prespecified patients with the same preoperative prolapse stage and uterus removal.

The major limitation is the retrospective nature and its relatively small sample size, which may limit the generalizability of our findings. Additionally, because most women in our study are sexual inactivity, it is impossible to evaluate the impact of the two procedures on sexual life. Finally, permanent suture use has potential risk of suture erosion into the vagina [23]. Therefore, randomized controlled trials with large-scale, multi-center and rigorous design are planned to further validate the effectiveness and safety of this approach. Moreover, evaluation of suture-related complications of the CSS technique in sexually active women will be explored.

## Conclusion

Compared with the conventional anterior colporrhaphy, the present novel CSS technique provided better results in anatomical recovery with low rate of postoperative morbidity, indicating that it can be recommended as an alternative method for anterior vaginal prolapse.

## Data Availability

The datasets used and/or analyzed during the current study are available from the corresponding author on reasonable request.

## Abbreviations

POP: Pelvic organ prolapse
AC: Anterior colporrhaphy
CSS: Cable-suspended suture
POP-Q: Pelvic organ prolapse quantification
PFDI-20: Pelvic Floor Distress Inventory-20
QOL: Quality of life
PFIQ-7: Pelvic Floor Impact Questionnaire-7
SLF: Sarcrospinous ligament fixation
SUI: Stress urinary incontinence
TVT: Tension free vaginal tape
ATFP: Arcus tendinous fascia pelvis
